# Seroprevalence of influenza A virus in pigs and low risk of acute respiratory illness among pig workers in Kenya

**DOI:** 10.1186/s12199-019-0808-6

**Published:** 2019-08-17

**Authors:** Eric Mogaka Osoro, Shirley Lidechi, Doris Marwanga, Jeremiah Nyaundi, Athman Mwatondo, Mathew Muturi, Zipporah Ng’ang’a, Kariuki Njenga

**Affiliations:** 1 Jomo Kenyatta University of Agriculture and Technology, Nairobi, Kenya; 2 Kenya Medical Research Institute, Nairobi, Kenya; 3 Ministry of Health, Nairobi, Kenya; 4 Ministry of Agriculture and Irrigation, Nairobi, Kenya; 5 Washington State University, Pullman, USA

**Keywords:** Acute respiratory illness, Influenza A virus, Pig workers, Zoonoses

## Abstract

**Background:**

Influenza A viruses pose a significant risk to human health because of their wide host range and ability to reassort into novel viruses that can cause serious disease and pandemics. Since transmission of these viruses between humans and pigs can be associated with occupational and environmental exposures, we investigated the association between occupational exposure to pigs, occurrence of acute respiratory illness (ARI), and influenza A virus infection.

**Methods:**

The study was conducted in Kiambu County, the county with the highest level of intensive small-scale pig farming in Kenya. Up to 3 participants (> 2 years old) per household from pig-keeping and non-pig-keeping households were randomly recruited and followed up in 2013 (Sept-Dec) and 2014 (Apr-Aug). Oropharyngeal (OP) and nasopharyngeal (NP) swabs were collected from participants with ARI at the time of study visit. For the animal study, nasal and oropharyngeal swabs, and serum samples were collected from pigs and poultry present in enrolled households. The human and animal swab samples were tested for viral nucleic acid by RT-PCR and sera by ELISA for antibodies. A Poisson generalized linear mixed-effects model was developed to assess the association between pig exposure and occurrence of ARI.

**Results:**

Of 1137 human participants enrolled, 625 (55%) completed follow-up visits including 172 (27.5%) pig workers and 453 (72.5%) non-pig workers. Of 130 human NP/OP swabs tested, four (3.1%) were positive for influenza A virus, one pig worker, and three among non-pig workers. Whereas none of the 4462 swabs collected from pig and poultry tested positive for influenza A virus by RT-PCR, 265 of 4273 (6.2%) of the sera tested positive for virus antibodies by ELISA, including 11.6% (230/1990) of the pigs and 1.5% (35/2,283) of poultry. The cumulative incidence of ARI was 16.9% among pig workers and 26.9% among the non-pig workers. The adjusted risk ratio for the association between being a pig worker and experiencing an episode of ARI was 0.56 (95% CI [0.33, 0.93]), after adjusting for potential confounders.

**Conclusions:**

Our findings demonstrate moderate seropositivity for influenza A virus among pigs, suggesting the circulation of swine influenza virus and a potential for interspecies transmission.

## Background

Influenza A viruses pose a significant risk to human health because of their wide host range (swine, birds, horses, dogs, cats, sea mammals) and capacity to reassort into novel viruses that can cause serious epidemics or pandemics [1]. Pigs are believed to play an important part in the evolution of viruses of pandemic potential because of their inherent ability to allow replication of swine, avian, and human influenza viruses and potential to have mixed infections [2–4]. For example, the 2009 influenza A H1N1 pandemic virus that was associated with 150,000–570,000 deaths globally was the product of re-assortment of circulating human influenza and avian influenza strains with pigs suspected as the mixing vessel [5].

Pig-to-human and human-to-pig influenza (reverse zoonosis) virus transmission events have been documented in North America, Europe, Asia, and Africa [6–11]. Severe disease following these zoonotic events has been reported in persons with chronic medical conditions, although most such infections are mild or subclinical [12, 13]. Reverse zoonosis of influenza virus is considered an important source of swine influenza virus (SIV) diversity which reduces the efficacy of vaccines to SIV in pigs [14].

The transmission of influenza viruses between pigs and humans is not only associated with occupational and environmental exposures, but also with the virus evolution and emergence of novel transmissible strains capable of infecting humans and spreading from person to person that can lead to pandemics [6, 8, 15].

Studies have shown evidence of infection with newly emerging SIVs as well as higher prevalence of SIVs among persons whose occupation involves close contact with pigs [6, 16]. Findings from a preliminary study in pigs from a Kenyan slaughterhouse revealed an overall influenza A seroprevalence of 15%, including > 12% seroprevalence of the pandemic 2009 H1N1 influenza virus, suggesting transmission of influenza viruses from humans to pigs [17].

The growing demand for pig products in Kenya has resulted in rapid growth in intensive small-scale pig farming [18]. Although pig workers in such livestock production systems may be exposed to swine influenza viruses, no studies on the occupational exposure risks to influenza viruses have been documented in the country.

Here, we conducted a longitudinal study to determine the association between occupational exposure to pigs and the occurrence of ARI and influenza A virus infection. We monitored ARI among the humans to determine its utility in detection of influenza virus infections among pig workers. We also assessed the farming practices associated with high risks of influenza virus transmission among pig keepers.

## Methods

### Study area

**Fig. 1:**
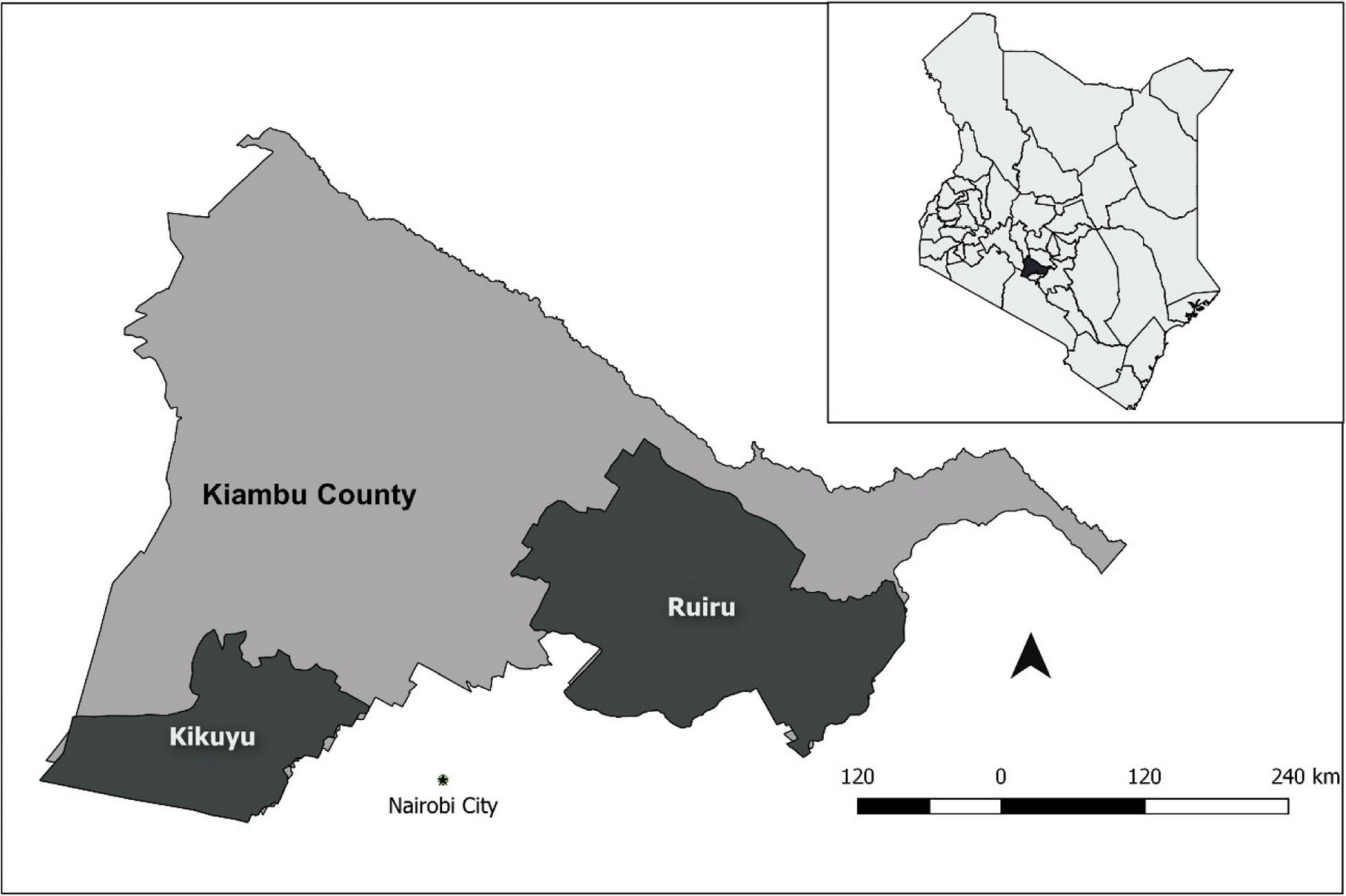
Map of Kiambu County showing the selected administrative locations where households were sampled. The households were sampled from within two sub-counties of Kiambu County—Kikuyu and Ruiru. The inset is a map of Kenya with Kiambu County highlighted in dark color

The study was conducted in Kiambu County in Kenya (Fig. 1), a county that has the largest proportion of intensive small-scale pig farmers in Kenya [18]. Within the county, we selected two sub-counties that have the highest number of pig farms and selected households based on whether they kept pigs or not.

Pig-keeping households were selected by systematic random sampling from a comprehensive list of pig farmers in the area prepared by the local veterinary officers. Two to three non-pig-keeping households were selected from the neighbourhood of each selected pig-keeping household.

### Study design and sampling

The participants from pig-keeping and non-pig-keeping households were enrolled in September 2013 and April 2014, with a follow-up visit conducted 12 to 14 weeks after enrollment. Concurrent cross-sectional sampling of pigs and poultry in enrolled households was conducted at enrollment and follow-up visits.

All members above 2 years of age in selected households were eligible for enrollment. In each household, up to 3 persons were randomly selected. At enrollment, a questionnaire was administered and nasopharyngeal (NP) and oropharyngeal (OP) swabs collected from participants who met the acute respiratory illness (ARI) case definition.

The participants were then visited after 12 to 14 weeks to administer the follow-up questionnaire as well as collect NP/OP swabs if the participant had ARI at the time of follow-up. Pig exposure was defined as cleaning barns, feeding, or slaughtering pigs as part of routine daily activities (> 3 times a week) for the month preceding the study interview. Poultry exposure was similarly defined for those working with poultry.

Participants with pig or poultry exposures were classified as pig workers and poultry workers, respectively. The determination of pig or poultry exposure status was made during enrollment. Acute respiratory illness (ARI) was defined as an illness with a history of fever or cough lasting less than 10 days.

Animal samples were collected from pigs and poultry on the farm at enrollment and follow-up. Pig nasal swabs and sera were collected from all age groups including piglets, weaners, growers, finishers, sows, and boars. The number of pigs sampled was proportionate to herd size with all animals sampled from small herds (< 10 pigs) and up to 15 animals sampled from herds with > 10 pigs.

For poultry, sera and oropharyngeal swabs were collected from up to 3 animals each of chickens, ducks, turkeys, and geese present on the farms.

### Sample size

The primary outcomes on the human component of the study were the prevalence of influenza A virus infection and the number of ARI episodes reported during the follow-up period.

The sample size was calculated assuming 8% prevalence of acute respiratory illness among pig workers (exposed), and 2% among non-pig workers (unexposed) translating to a sample size of 394 participants with 99 in the exposed and 295 in the unexposed group (exposed to unexposed ratio of 1:3) [19, 20].

In the animal study, the primary outcome was the detection of influenza A virus infection and seroprevalence of influenza A virus. A minimum sample size of 392 pigs was determined based on an expected seroprevalence of 15% and a design effect of two at 95% confidence level.

### Sample collection and testing

The NP and OP swabs were collected from each enrolled human participant that met the case definition of ARI at the time of sampling. The two swabs were put together in a cryovial viral transport media (VTM), temporarily stored in a cool box at 2–8 °C, and later in the day transported to the Kenya Medical Research Institute (KEMRI) laboratory in Nairobi and stored at – 80 °C until testing. Serum, and nasal or OP swabs were collected from each pig and poultry. Each animal swab was separately placed in a cryovial containing VTM, temporarily stored in a cool box at 2–8 °C and transported to KEMRI laboratory in Kisumu for storage at – 80 °C until testing.

Animal serum was tested for antibodies against influenza A viruses using the IDEXX® ELISA (FlockChek AI MultiS-Screen Ab Test Kit®, Westbrook, Maine), following the manufacturer’s instructions [21, 22]. A seropositive herd was defined as any household with at least one IgG antibody positive pig.

Human NP/OP samples were tested for viral RNA by real-time reverse transcriptase polymerase chain reaction (RT-PCR) using CDC primers and probes for influenza A and influenza B viruses [22, 23]. The RNA was extracted using the QIAamp RNA extraction kit (Qiagen Inc., Valencia, CA) following the manufacturer’s instructions. Cutoff for positivity was read at cycle threshold (C_T_) values ≤ 40. Positive and negative controls were used to validate the test assay.

Subtyping was attempted for influenza A positive swabs for seasonal human influenza, avian, and swine influenza [24]. Animal nasal and OP swabs were screened for influenza A virus by RT-PCR using the CDC protocol for influenza A virus detection that targets the matrix gene [23].

### Data collection and analysis

Standardized questionnaires on smartphones were administered to all participants to collect data on demographics, clinical symptoms, and exposure to risk factors including specific activities with reference to pig and poultry raising, transportation, slaughtering, and dressing. For animals, data on herd demographics and risk factors (age, species present on the farm, herd size, species raised, and husbandry practices) were collected.

We used R Statistical Software (version 3.5.1) for data cleaning and analysis [25]. Descriptive statistics were determined for socio-demographic and other characteristics comparing pig workers and non-pig workers. Categorical variables were compared using chi-square test and Fisher’s exact test while continuous variables were compared using the Student *t* test. The cumulative incidence for ARI was calculated as the number of episodes reported by participants divided by the total number of participants.

Crude risk ratios were determined for the initial assessment of the association between pig exposure and episodes of ARI. We applied the generalized linear mixed model (GLMM) using the Poisson distribution to adjust the risk ratio between pig exposure and ARI for clustering and potential confounding. We assessed for overdispersion before applying the Poisson distribution where a *p* value of < 0.05 would indicate overdispersion. The predictor variables (fixed effects) included in GLMM to predict the occurrence of ARI episodes were pig workers, age, sex, occupation, education level completed, reported chronic disease, and poultry exposure. We accounted for clustering at household and individual level (repeat ARI episodes) by including the variables as random effects in the mixed model. The GLMM was done using the lme4 package in R statistical software where the estimation is based on the maximum likelihood [26].

Model selection was conducted using stepwise selection using Akaike information criterion and Bayesian information criteria measures where lower values suggest a better model fit [27].

The adjusted risk ratio and the 95% confidence intervals were then computed and a *p* value of < 0.05 considered statistically significant.

### Ethical considerations

The study was approved by the KEMRI Scientific and Ethics Review (Protocol # SSC 2557), KEMRI Animal Care and Use Committee, the CDC Institutional Review Board and National Institute of Health, and Division of Microbiology and Infectious Diseases review board. Informed consent was obtained from all participants. Assent and parental permission were obtained for minors.

## Results

### Household characteristics

**Fig. 2:**
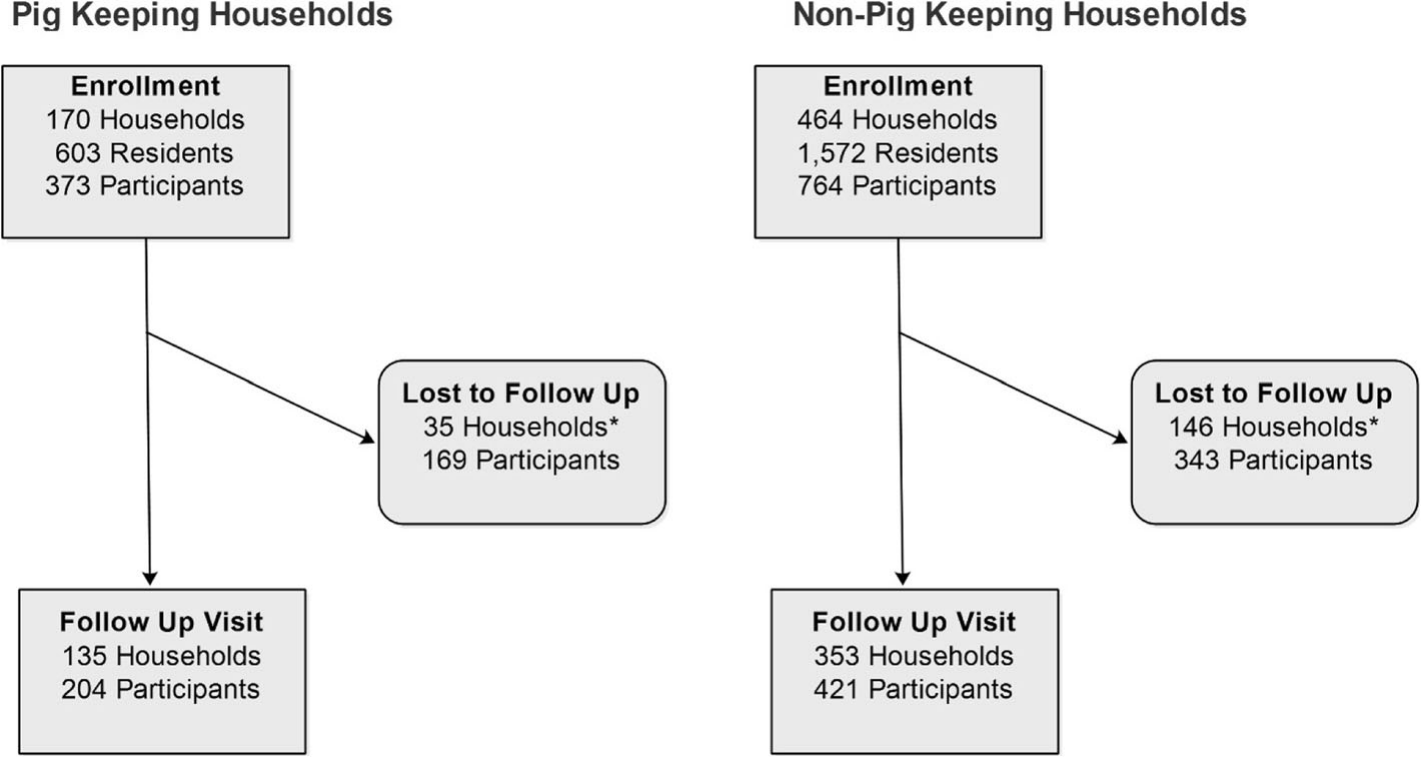
Schema of households’ and participants’ enrolment, follow-up, and lost to follow-up. Participants from pig-keeping and non-pig-keeping households were enrolled and had a follow-up visit after 12 or 14 weeks. *Only households where no participant was available during the follow-up were included

A total of 634 households with 2175 persons were enrolled, of which 488 (77.0%) households participated in the follow-up visit. From 170 pig-keeping households, 373 (61.9%) participants were enrolled, of which 204 (54.7%) had a follow-up visit. From 464 non-pig-keeping households, 764 (48.6%) participants were enrolled, of which 421 (55.1%) had a follow-up visit (Fig. 2). The average number of participants enrolled per household was 2.2 and 1.7 for pig-keeping and non-pig-keeping households, respectively. There were no significant differences in sex, age, or education level completed of household heads between the pig-keeping and non-pig-keeping households.

**Fig. 3:**
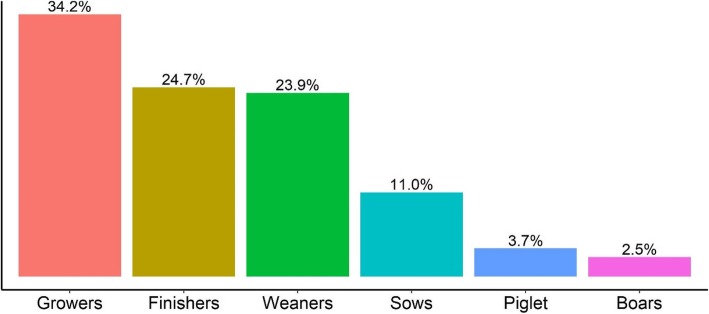
Proportion of pigs sampled (*n* = 2066) in pig-keeping households by maturity status, Kiambu, 2013–2014

Nearly all (97.9%) pig-keeping households reared other animals, significantly higher than 73.1% in non-pig-keeping households (*p* < 0.001). Among pig-keeping households, the median number of pigs was 13 (range 1 to 200) and 54.2% had reared pigs for at least 2 years. A total of 2066 pigs were sampled, of which 1118 (63.2%) were female and nearly half (48.6%) were either finishers or growers (Fig. 3). Besides pigs, the majority of the pig-keeping farms reared poultry (83.3%).

### Individual characteristics

Of 1137 participants enrolled, 625 (55%) had a follow-up visit and included in the analysis (Fig. 2). There were no significant differences in sex, occupation, and highest education level completed between the participants who received follow-up visits and those lost to follow-up (*p* > 0.05). The demographic characteristics and pig worker status of the 625 participants who had follow-up visits are presented in Table 1. Among the 172 pig workers, 92.4% were residents of pig-keeping households, 55.2% were male, and the 21–40 years age group accounted for 45% of the participants. About 80% of the pig workers reported their occupation as farming, 80% were also poultry workers with 2.3% reporting no formal education (Table 1). Non-pig workers were mostly female (57.2%), about one third (34.4%) were between 21 and 40 years old and 54.1% were farmers (Table 1). Human influenza vaccination in the previous 12 months was reported by two pig workers and one non-pig worker.

**Table 1:** Demographic and other characteristics of study participants by pig worker status, Kiambu, 2013–2014

Variable	Pig workers (*N* = 172), *n* (%)	Non-pig workers (*N* = 453), *n* (%)	*p* value
Pig-keeping household			
Yes	159 (92.4)	45 (9.9)	< 0.001
No	13 (7.6)	408 (90.1)	
Follow-up month			
Sept–Dec	95 (55.2)	224 (49.4)	0.229
Apr–Aug	77 (44.8)	229 (50.6)	
Sex			
Female	77 (44.8)	259 (57.2)	0.007
Male	95 (55.2)	194 (42.8)	
Age in years, mean (SD)	40.3 (15.7)	39.6 (18.6)	0.646
Age group, years^a^			
Below 10	0 (0.0)	20 (4.4)	0.005
10 to 20	15 (8.8)	67 (14.9)	
21 to 40	77 (45.0)	155 (34.4)	
41 to 60	56 (32.7)	144 (31.9)	
Above 60	23 (13.5)	65 (14.4)	
Highest level of education completed^a^			
No formal Education	4 (2.3)	13 (3.0)	0.615
Primary	68 (39.8)	173 (39.3)	
Secondary	68 (39.8)	157 (35.7)	
Post-secondary	31 (18.1)	97 (22.0)	
Occupation^a^			
Business	4 (2.5)	49 (13.5)	< 0.001
Farmer	135 (82.8)	196 (54.1)	
Office Worker	10 (6.1)	33 (9.1)	
Unemployed	14 (8.6)	84 (23.2)	
Poultry worker^a^			
Yes	136 (79.5)	250 (55.4)	< 0.001
No	35 (20.5)	201 (44.6)	
Use tobacco^a^			
Yes	16 (9.3)	34 (7.5)	0.566
No	155 (90.7)	419 (92.5)	
Reported chronic disease^a^			
Yes	23 (13.4)	81 (17.9)	0.214
No	149 (86.6)	371 (82.1)	

^a^Variable has some missing data. *SD* standard deviation

### Human and animal influenza A virus results

A total of 130 swab samples were collected from human participants who reported ARI either at enrolment or during follow-up with only five participants having samples collected at both enrolment and follow-up visits.

Among 91 samples from participants who completed the follow-up visit, 24 (26.4%) were from pig workers and 67 (73.6%) from non-pig workers. Four (3%) human swabs tested positive for influenza A by RT-PCR: one from a pig worker and three from non-pig workers. The positive samples could not be subtyped.

A total of 4462 nasal and oropharyngeal swabs from animals were collected; 2173 (48.7%) from chicken, 2066 (46.3%) from pig, 126 (2.8%) from ducks, 56 (1.3%) from geese, and 41 (0.9%) from turkey. None of the swabs was positive for influenza A virus by RT-PCR. A total of 4273 serum samples were collected from the animals, including 2283 (53.4%) from poultry and 1990 (46.6%) from pigs. Overall, 265 (6.2%) of the animal sera were positive for influenza A virus by ELISA, including 230 of 1990 (11.6%) pig sera and 35 of 2283 (1.5%) poultry sera. Among poultry, the seropositivity for influenza A was 3.3% for geese, 2.9% for ducks, 1.4% for chicken, and 0% for turkeys.

Fifty-eight (34.1%) of the pig-keeping households had at least one seropositive pig during the sampling points. The median number of seropositive pigs among these households was two (range 1 to 10).

### Association between pig workers and acute respiratory illness

We examined the risk of occurrence of ARI by pig worker status during the follow-up period. Overall, a total of 151 episodes of ARI were reported from 116 participants, giving a combined cumulative incidence of 24.2%. Three-quarters (87) of the participants with ARI reported only one episode, 26 (22.4%) reported two episodes, and three (2.6%) reported between three and five episodes.

Among pig workers, there were 29 ARI episodes (cumulative incidence of 16.9%) while among non-pig workers there were 122 ARI episodes (cumulative incidence 26.9%). On bivariate analysis, pig workers had a 47% lower risk of having ARI compared to non-pig workers with an unadjusted risk ratio (RR) of 0.53 (95% CI [0.33, 0.84]).

A Poisson generalized linear mixed model was used to adjust the RR for potential confounding against age, sex, poultry exposure, education, month of sampling, occupation, and reported chronic disease. The adjusted RR for pig workers was 0.56 (95% CI [0.33, 0.93]), indicating that pig workers had a 44% lower risk of having ARI compared to non-pig workers (Table 2).

**Table 2:** Multivariate Poisson generalized mixed-effects model for the association between occurrence of acute respiratory infection and pig worker status, Kiambu, 2013–2014

Exposure/potential confounder	Crude RR (95% CI)	Adjusted RR (95% CI)	*p* value for adjusted RR
Pig worker	0.53 (0.33, 0.84)	0.56 (0.33, 0.93)	0.025
Age in years	1.00 (0.99, 1.01)	0.99 (0.98, 1.00)	0.172
Reported chronic disease	1.48 (0.93, 2.35)	1.52 (0.88, 2.62)	0.134
Education level completed			
Primary	0.53 (0.25, 1.12)	0.35 (0.15, 0.86)	0.022
Secondary	0.4 (0.19, 0.86)	0.31(0.12, 0.78)	0.013
Post-secondary	0.59 (0.27, 1.33)	0.41 (0.16, 1.08)	0.071
Household member with ARI in previous 3 months	3.13 (2.03, 4.83)	2.97 (1.78, 4.93)	< 0.001

The model also showed that participants from households where members had reported acute respiratory illness in the previous 3 months had three times higher risk of reporting ARI.

Participants who had completed primary or secondary education had about a 60% lower risk of developing ARI compared to those without formal education (Table 2).

### Assessing risky practices that promote transmission at the human-animal interface

To assess risky practices associated with the transmission of the influenza virus at the human-animal interface, we restricted the analyses to pig-keeping households. The majority (88%) of the households had pens with concrete floors, with 30% of them using sawdust for beddings.

While the majority of the households fed the pigs with commercial feeds, about 60% of the households also used scraps/wastes from the household or the market.

Less than half (46%) of the households separated new pigs before allowing them to mix with the rest of the herd, and 11% of the households had added new pigs to the herds within the month preceding the interview. Among households which practised quarantine for new pigs, about half (52.5%) quarantined for 3 days or less. Almost three-quarters of the pig-keeping households did not vaccinate their pigs for any disease, and none of the households reported vaccinating the pigs against influenza. A quarter of the households reported that the pigs regularly mixed with other animals on their farm (or animals in other farms).

Overall, there was low usage of personal protective equipment by pig workers, with 96.3% using eye protection less than once a week and less than half (45.7%) using protective coveralls and aprons when working in pig pens.

However, 72.2% used footwear most of the time (> 5 times a week) and nearly all (96.9%) reported washing hands after working in the barns

## Discussion

This study documents a human-animal environment in Kiambu County with robust influenza virus circulation, demonstrated by > 6% seropositivity of the virus among the pig and poultry populations including almost 12% in the pig population.

Similarly, we found 3% of humans with acute respiratory illness positive for influenza A virus by RT-PCR. These findings agree with other studies, including a Kenyan study that reported 15% influenza virus prevalence among pigs and other studies elsewhere in Africa and Asia reporting as high as 67% influenza virus seroprevalence among swine in live markets [28–31].

Since swine influenza vaccination was not practiced by the farmers in our study, the level of seropositivity suggests exposure to circulating virus. The 1.4–3.3% prevalence of influenza virus among the poultry species (chicken, ducks, and geese) reported in our study support the finding that Kiambu County is an environment of animal influenza virus circulation.

Our finding of 3% influenza positive ARI human cases is lower than a community study in Romania that reported 13% influenza A virus positive samples among ARI cases by PCR [32, 33]. The low number of PCR positives among ARI cases could be due to sampling a healthy population who did not have active infection at the time of sampling or had a subclinical infection. The follow-up time per participant was about 3 months and seasonality of influenza could account for the relatively low number of positive cases.

We found that pig workers had about half the risk of having ARI compared to non-pig workers. This could be due to healthy worker effect where persons who work closely with pigs have systematic differences associated with occurrence of ARI compared to the non-pig exposed [34].

For example, pig workers had a lower proportion of < 20-year-olds compared to non-pig workers (9% vs 19%, respectively). Our study finding is contrary to other studies that showed pig workers have higher odds of respiratory illness compared to non-pig workers [35, 36]. However, these were cross-sectional studies conducted in high-income countries to assess chronic respiratory health among farmers. It is likely that serology results from our study would have been higher in pig workers, or at least comparable with those of non-pig workers. However, attempts to carry out serology in humans were unsuccessful.

Our finding of lower risk of ARI among pig workers suggest that monitoring ARI in the general population would likely miss potential zoonotic events. Zoonotic influenza events are likely to first appear among those working or exposed to pigs or poultry, mostly young adults. With the reported levels of exposure to swine influenza among pigs in our study, including the pig workers in the influenza surveillance could enhance efforts to detect early zoonotic influenza events.

Focused surveillance in an occupationally exposed group offers a potentially cost-effective mechanism to monitor trends of influenza, including influenza zoonotic events. A number of innovative and affordable approaches such as mobile-based surveillance could be applied to offer the needed early warning mechanism to identify increases in acute or severe respiratory episodes in this group [28].

In the mixed-effects model for occurrence of ARI, having a household member with an episode of ARI in the previous 3 months was an independent risk factor. These findings are consistent with the known transmission of pathogens associated with ARI through close contact [37].

When we assessed the farming practices associated with increased transmission of zoonotic influenza, we found that pig-keeping households also kept poultry (mixed animal farming), and majority did not quarantine newly introduced pigs before mixing with the herd [30]. In addition, there was inadequate use of personal protective equipment while working in pig pens. Studies have documented that lack of quarantine and uncontrolled movement between farms, and poor usage of personal protective equipment are risk factors for influenza transmission [29, 31]. These transmission-promoting practices could be due to the lack of knowledge and facilities for biosecurity measures.

This study had several limitations. We had a loss to follow-up of 45% of the enrolled participants. While there were no differences in age or education level between those lost to follow-up and those retained, they could have had different experiences on ARI, influenza A positivity and farming practices.

Although the study period included 9 months of the year, follow-up period for each participant was about 3 months. It is also likely that not all ARI episodes were reported due to participant recall bias. A longer and more frequent follow-up and with serological testing would allow for sampling to account for seasonality of influenza infections as well as subclinical cases. Another limitation is that we were not able to conduct hemagglutination inhibition (HI) tests to determine the influenza A virus strains circulating among pigs. However, a study in Kenya in 2012 [38] reported 72% of seropositive pigs had influenza virus (A/H1N1/pdm09) by HI, findings that could reflect the influenza virus strains among pigs in our study.

## Conclusion

Our study documents moderate seropositivity among pigs for influenza A virus, suggesting circulation of swine influenza virus and therefore a potential for interspecies transmission. Swine workers had a lower risk of ARI compared to non-swine workers. While serological studies among swine workers may be a better approach to quantify the risk of zoonotic influenza infection, focused syndromic surveillance in this population offers an important early warning system for such zoonotic events in Kenya.

## Data Availability

The datasets used and/or analyzed during the current study are available from the corresponding author on reasonable request

## Abbreviations

ARI: Acute respiratory illness
CDC: Centers for Disease Control and Prevention
CI: Confidence interval
ELISA: Enzyme-linked immunosorbent assay
GLMM: Generalized linear mixed model
KEMRI: Kenya Medical Research Institute
NP: Nasopharyngeal
OP: Oropharyngeal
RT-PCR: Real-time reverse transcriptase polymerase chain reaction
SD: Standard deviation
SIV: Swine influenza viruses
VTM: Virus transport media
